# Polygenic Sex Determination System in Zebrafish

**DOI:** 10.1371/journal.pone.0034397

**Published:** 2012-04-10

**Authors:** Woei Chang Liew, Richard Bartfai, Zijie Lim, Rajini Sreenivasan, Kellee R. Siegfried, Laszlo Orban

**Affiliations:** 1 Reproductive Genomics Group, Temasek Life Sciences Laboratory, Singapore, Singapore; 2 School of Biological Sciences, Nanyang Technological University, Singapore, Singapore; 3 Department of Biological Sciences, National University of Singapore, Singapore, Singapore; 4 Department of Genetics, Max-Planck-Institut für Entwicklungsbiologie, Tübingen, Germany; 5 Department of Animal Sciences and Animal Husbandry, University of Pannonia, Keszthely, Hungary; Universitat Pompeu Fabra, Spain

## Abstract

**Background:**

Despite the popularity of zebrafish as a research model, its sex determination (SD) mechanism is still unknown. Most cytogenetic studies failed to find dimorphic sex chromosomes and no primary sex determining switch has been identified even though the assembly of zebrafish genome sequence is near to completion and a high resolution genetic map is available. Recent publications suggest that environmental factors within the natural range have minimal impact on sex ratios of zebrafish populations. The primary aim of this study is to find out more about how sex is determined in zebrafish.

**Methodology/Principal Findings:**

Using classical breeding experiments, we found that sex ratios across families were wide ranging (4.8% to 97.3% males). On the other hand, repeated single pair crossings produced broods of very similar sex ratios, indicating that parental genotypes have a role in the sex ratio of the offspring. Variation among family sex ratios was reduced after selection for breeding pairs with predominantly male or female offspring, another indication that zebrafish sex is regulated genetically. Further examinations by a PCR-based “blind assay" and array comparative genomic hybridization both failed to find universal sex-linked differences between the male and female genomes. Together with the ability to increase the sex bias of lines by selective breeding, these data suggest that zebrafish is unlikely to utilize a chromosomal sex determination (CSD) system.

**Conclusions/Significance:**

Taken together, our study suggests that zebrafish sex is genetically determined with limited, secondary influences from the environment. As we have not found any sign for CSD in the species, we propose that the zebrafish has a polygenic sex determination system.

## Introduction

Sex determination (SD) establishes the sexual fate of an organism and initiates the gonad differentiation process (reviews: [1], [2], [3]). A variety of signals, including genetic, environmental or even social cues, were found to be sex determinants in vertebrates (see reviews [4], [5], [6]).

The most extensively studied mode of genetic SD is chromosomal sex determination (CSD) as in mammalian and avian species, for example. In this system, sex is determined by a primary switch located on one or both members of a well-differentiated sex chromosomal pair (see e.g. [7], [8], [9], [10]). Since in mammals, including humans, sex is determined by CSD, the vast majority of our knowledge on the molecular regulation of vertebrate sex is based on data collected from such systems.

In the other type of genetic sex determination system, called polygenic (multigenic or multifactorial) sex determination (PGSD), the genes with strong influence on sex determination and/or gonad differentiation are distributed throughout the genome and the combination of their alleles determines the sex of the individual [11], [12]. This form of sex determination has not been studied extensively at the experimental level (for exceptions, see e.g. [13], [14]): European seabass [15] and a handful of cichlid species from Lake Malawi [16] are the only fish species that were shown to utilise this system to date.

Sex can also be determined by signals from the environment and there are several environmental effects known to influence sex of an organism. Temperature is one of the most commonly studied environmental cues for sex determination. In many reptiles, sex is determined by environmental temperature during the thermosensitive periods of embryo development or egg incubation (for reviews see [17], [18]). Examples of other environmental cues that also have an influence on sex include pH in guppy [19] and social interactions in some reef fishes [20].

Over the past decades, zebrafish (*Danio rerio*) has become an important laboratory model organism for many areas of research (for examples see e.g.: [21], [22], [23], [24], [25]). Despite being a popular model for developmental biology and biomedical research, very little is known about its sexual development (for review see [26]). Moreover, most of the current knowledge on zebrafish sexual development is related to its gonad differentiation (for reviews see [27], [28]) while the mode of its sex determination is still disputed.

Most cytogenetic studies showed that the zebrafish has chromosomes of similar size and morphology. This lack of distinct morphological differences together with poor karyotype banding pattern resulted in difficulties with accurately assigning chromosomal pairs (for review see [29]). Therefore, it is not easy to search for sex chromosomes based on size differences, their distinct trademark in most mammalian and avian species. An alternative approach to cytogenetic approaches would be to search for differences between the two sexes at the level of the whole genome. PCR-based methods such as random amplified polymorphic DNA (RAPD; [30], [31]) and amplified fragment length polymorphism (AFLP; [32]) have been used successfully for identification of sex markers in fishes (see e.g. [33], [34], [35]) and other vertebrates (see e.g. [36], [37], [38]). Earlier, we have developed a new PCR-based mass genotyping technique called fluorescent motif enhanced polymorphism (FluoMEP; [39]) that combines the advantages of RAPD and AFLP. In this study, we have utilized this technology to search for sex-linked DNA markers in the genome of three fish species, including zebrafish.

Another molecular tool used for comparing the male and female zebrafish genome in this study is array comparative genome hybridization (aCGH). This method allows for the detection of differences, called copy number variations (CNV) [40], [41], between two complex DNA samples on a genome-wide scale [42], [43]. The method is based on hybridization of two samples onto a ‘tiling array’ that contains probes scanning through the whole genome at regular intervals. Originally, aCGH was developed for the analysis of chromosomes aberrations in cancer cells [43], [44]. Over the years, this method has also been utilized for various purposes such as studying evolution [45], [46], understanding the impact of CNV on transcriptome [47] and isolation of molecular markers [48].

The aim of this study was to perform a detailed analysis on zebrafish sex determination, by combining the power of traditional and molecular technologies. Through analysis of sex ratios in a large number of families, we show that i) sex ratios vary among different families; ii) parental genotypes have a major effect on the sex ratio; and iii) one of the two sexes can be depleted through systematic selection in a few generations. Moreover, PCR-based screens and aCGH performed by a custom-designed tiling array were both unable to find general differences between the genome of the two sexes in two different zebrafish strains. The above data all point towards a genetic mechanism of sex determination and the lack of a chromosomal sex determination system in the zebrafish. We, therefore, propose that zebrafish sex determination is polygenic.

## Materials and Methods

### Fish stocks and tail fin samples

Experiments performed at Temasek Life Sciences Laboratory were approved by Temasek Life Sciences Laboratory Institutional Animal Care and Use Committee (approval ID: TLL(F)-10-001) and performed according to its guidelines. Experiments performed at Max-Planck-Institut für Entwicklungsbiologie were registered at Regierungspräsidium Tübingen (approval ID 35/9185.46) and carried out according to the Protection of Animals Act (Tierschutzgesetz) and its guidelines. Zebrafish (*Danio rerio*) of the AB strain, Tübingen strain and a wild type strain, called Toh, purchased from a local aquarium shop were used in this study. All zebrafish were kept in AHAB (Aquatic Habitats, Apopka, FL, USA) recirculation systems according to standard protocols [49], with the exception of the population density study in which fish were raised in an Aqua Schwartz system. Guppy (*Poecilia reticulata*) fin clips from visually sexed individuals were kind gifts from Dr. Rob Brooks (UNSW, Sydney, Australia). Visually sexed rosy barb (*Puntius conchonius*) individuals were purchased from a local fish trading company (Qian Hu Fish Farm, Singapore). Their tail fin samples were collected under anesthesia and stored in absolute ethanol at −20°C until use.

### Fish husbandry

Adult zebrafish were kept as mixed sex groups in 2.75 L tanks at a density of <10 individuals per liter. Breeding was carried out in meshed-bottom mouse cages of one liter volume placed into a second cage containing egg water. Breeding pairs were set up at the previous evening in the presence of artificial plants and eggs were collected before noon the next day. Pairs that were reluctant to yield eggs were given a slight cold shock by adding ice-cold egg water (about 20% of the tank volume) 1–2 hours after the start of breeding period. Ripe females that failed to produce eggs with two different males were gently squeezed to aid the removal of eggs, if any, potentially ‘stuck’ in their body and set up for repeated mating one week later.

Fertilized eggs were collected from the bottom of the cage, rinsed on a tea filter and transferred into plastic trays with egg water containing methylene blue. Survivals were recorded at 24 and 48 days post fertilization (dpf). Batches with survival below 50% during this period were discarded and their parents were crossed again later. Embryos were transferred onto the AHAB system before hatching and they were grown there at the following densities (unless indicated otherwise): <100/L for embryos, <80/L for larvae, <20/L for juveniles and <10/L for young adults.

### Sexing zebrafish

Zebrafish were sexed visually, based on the following two criteria (unless otherwise noted): i) general body shape; and ii) the presence of ‘genital papilla’ (or cloacal protrusion; [50]) in females (observed on unstressed fish kept in water). Individuals with intermediate body shape and poorly observable papilla were gently squeezed and checked for eggs or sperm. In absence of either, individuals were culled, dissected under a stereo microscope and their gonad was analyzed. Those individuals with unclear sex were not included in the calculation of sex ratio. Only 7 out of the 62 families sexed had such individuals and their ratio was typically less than 5%.

### Selection experiment for increased sex bias

We have performed a multi-generation selection experiment in order to increase sex bias. Based on the sex ratio of the offspring we have chosen five lines to be used for selection against males or females (Fig. S1). Pairwise full-sib crosses were performed according to a multifactorial design. The offspring were sexed at about 3 months of age and family sex ratio was recorded. From crosses that produced highly skewed sex ratio, usually three robust males and three robust females were chosen as brooders to produce the next generation.

### Population density effects on sex ratio

Rearing density experiments were performed using the Tübingen strain. Embryos and larva were raised at 29°C in petri dishes until 5 dpf at which time they were transferred to 1.5 L of fish water at varying densities. At 10 dpf, 600 ml of fish water was added to each tank to facilitate counting of larvae. At approximately 14 dpf, larval were placed into circulating water, resulting in 2 L of water per tank. From 5 dpf to about 14 dpf, larva were fed powdered fry food two times daily after which time the food source was changed to freshly hatched *Artemia nauplia*.

Two experiments were performed to test the effect of rearing density on sex ratios. In experiment 1, a total of 44 populations were analyzed spanning a period of about four months. In experiment 2, 30 populations were analyzed spanning about six and a half months. Embryos were collected from pairwise matings on day 0. In experiment 1, embryos from 2 or 3 crosses were often pooled, however more than one pool was often collected per day. Embryos were then sorted into petri dishes containing 50 embryos. In the second experiment, all embryos collected on a given day were pooled before sorting into dishes containing 50 embryos each. On day 5 dpf, larvae were set out at densities of 100, 50 or 25 larvae in 1.5 L of fish water. In experiment 2, the same number of tanks per density were set up out on a given day (e.g. 2 tanks with 100 larvae, 2 tanks with 50 larvae and 2 tanks with 25 larvae) whereas in experiment 1 the number of tanks per density per day was not controlled (Table S1 and Fig. S2). Overall, the difference in the design of the two experiments should have resulted in a lesser degree of genetic diversity between populations in experiment 2 compared to experiment 1. Larva and juvenile fish were counted every 10 days from 10 dpf up to 30 or 40 dpf for most (Table S1 and Fig. S2). In initial experiments, little to no lethality was observed after 30 dpf thus, in experiment 2 counting ceased after 30 dpf for most populations (Table S1 and Fig. S2). All fish were raised to adulthood and then sexed. For the first 30 populations, fish were sexed by dissection and observation of the gonad and subsequent populations were sexed based on coloration.

### FluoMEP assay

Genomic DNA (gDNA) samples were extracted from tail fins by digesting them at 55°C overnight in 800 µl of SET buffer (0.5% SDS, 50 mM EDTA, 10 mM Tris/Cl pH 8.0, 200 mM NaCl) and 250 µg/ml Proteinase K (Roche Diagnostics, Indianapolis, IN, USA). Then standard phenol chloroform extraction [51] was performed and the gDNA pellet was dissolved in 100 µL of 1× TE. Pooled male and female samples were generated by combining equal quantity of individual gDNA samples (nine individuals of rosy barb, eight individuals of guppy and four individuals of zebrafish for each sex). FluoMEP screening was carried out as described previously [39]. Bulk segregant analysis [52] was performed using the male and female pooled gDNA samples to screen for potentially sex-linked markers. Potential markers were then subjected to an additional round of analysis on the individuals that formed the pooled samples for confirmation.

### Array comparative genomic hybridization

Four families of zebrafish were used for aCGH and each family consisted of the parents, two male offspring and two female offspring individuals. Two of the families were from the AB strain and the other two were from the Toh strain. Genomic DNA samples for aCGH were extracted from tail fins using DNeasy Blood and Tissue kit (Qiagen) according to the manufacturer's instructions with slight modification. Instead of 2 hours incubation, tail fin samples were incubated overnight at 55°C in lysis buffer AL and proteinase K with slow shaking (70 rpm). The quality of extracted gDNA samples was checked on Nanodrop 1000 (Thermo Scientific, Waltham, MA, USA) and by agarose gel electrophoresis.

Individual samples were labelled with NimbleGen Dual-Color DNA Labelling Kit (Roche NimbleGen, Madison, WI, USA) and hybridization was carried out according to the manufacturer's instructions with MAUI hybridization system (BioMicro Systems, Salt Lake City, UT, USA). The oligo array was custom-designed by NimbleGen (Roche NimbleGen) based on zebrafish Zv7 (danre5) genome assembly. During the course of this study Zv8 (danre6) was released, all probes were re-mapped onto the new assembly for data analysis. Each array contained 120 thousand probes (55–70mers) with median spacing of about 10 kb. As preliminary tests have confirmed the accuracy of our procedure, no technical replicates were used for reasons of cost-efficiency.

The array was scanned at 5 µm resolution with Axon GenePix 4000B Microarray scanner (Molecular Devices, Sunnyvale, CA, USA). Raw fluorescent intensity data was retrieved by NimbleScan software (Roche NimbleGen) then imported into Partek Genomic Suite software (Partek Incorporated, St. Louis, MO, USA) for analysis. For copy number detection the genomic segmentation algorithm was used. A minimum of 5 markers were specified, *P* value threshold set at 0.001 and signal-to-noise ratio set at 0.3. All data was collected according to MIAME guidelines and deposited in NCBI GEO (GSE34338).

### Validation of aCGH results

For validation, we used the same gDNA samples that were analyzed by aCGH, plus an additional male and female offspring per family were included. All PCR primers (see Table S2 for primer sequences) were designed to target a sub-region of the copy number variable region (CNVR) using Primer3 version 0.4.0 [53]. Quantifast Probe PCR kit (Qiagen) was used for PCR validation of CNVR2 and CNVR5. The amplified products were then analysed by 2% agarose gel. A single-copy exon (DrSC23) was used as reference. For validation of CNVR3, real time quantitative PCR was carried out as described previously [54] using MyIQ real-time PCR detection system (Biorad, Hercules, CA, USA) with IQ Sybr Green Supermix (Biorad). Samples were normalized against two reference loci (DrSC19 and DrSC23), both of which were found to be a single-copy exon [55]. Relative quantification was calculated to estimate gain or loss of copy number with reference to paternal gDNA sample [54].

## Results

### Wide-ranging sex ratios among zebrafish families

**Figure 1:**
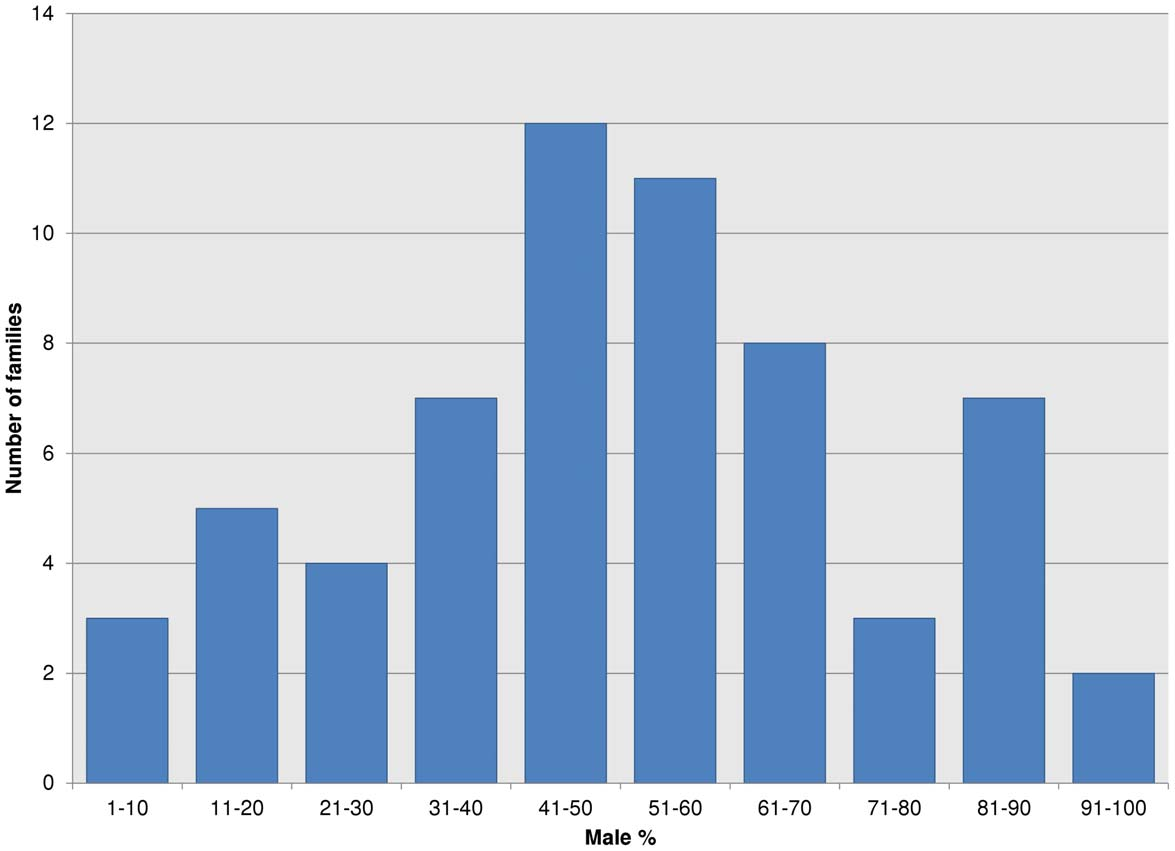
Wide-ranging sex ratios were observed among 62 zebrafish families. We have crossed randomly picked zebrafish individuals, grown their offspring to sexual maturity and determined their sex ratio based on presence/absence of sexual dimorphic phenotypic markers.

The classical method to determine if a species is using chromosomal sex determination system is to analyze the sex ratio among many families [5]. In the presence of strong CSD, the sex ratio is expected to be close to 50% [5]. In order to elucidate whether CSD is the main sex determination system in zebrafish, the sex ratios of 62 families were analyzed. The percentage of males among the families analyzed ranged from 4.8% to 97.3% with median of 51% (std. dev. ±22.6%; Fig. 1). Such a wide-ranging sex ratio among the families would be highly unusual for a predominantly sex chromosomal system.

### Skewed family sex ratios are very likely due to genetic factors

**Figure 2:**
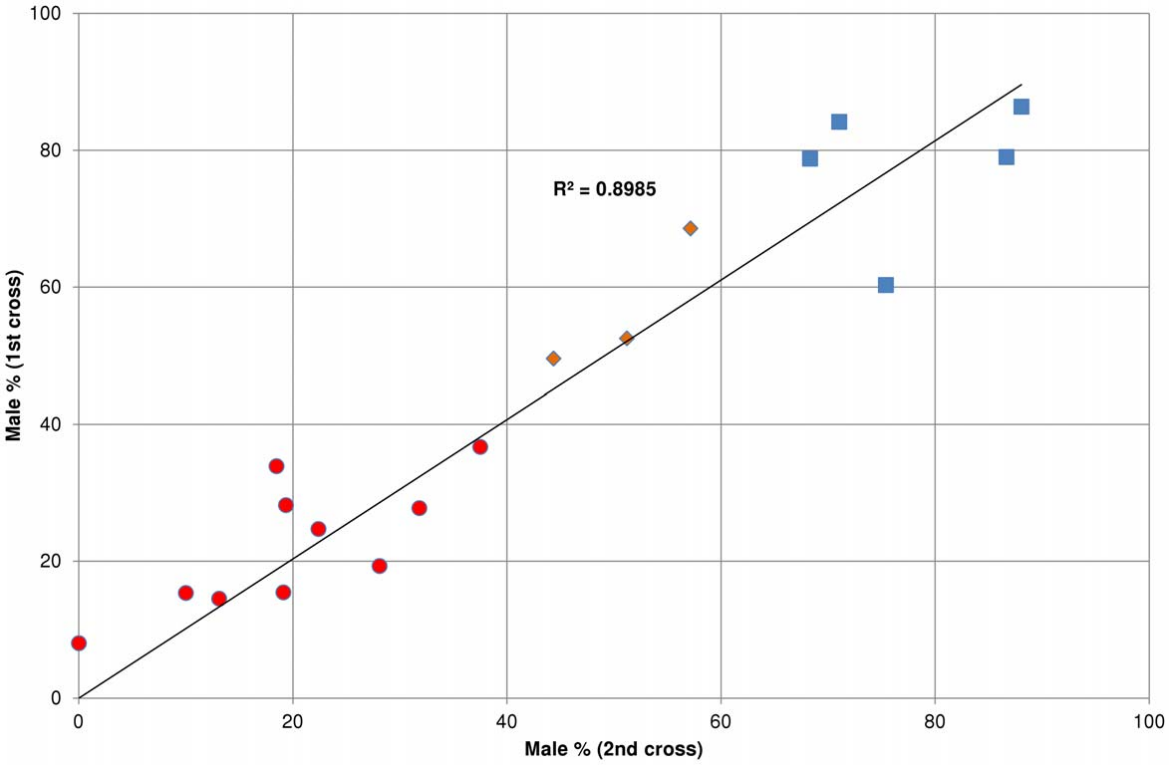
Sex ratios of offspring groups generated by repeated single pair crossings show close correlation. Nineteen randomly selected breeding pairs were crossed twice; eighteen of them are shown here. The high R^2^ value indicates that sex ratios between 1st and 2nd crosses from the same breeding pair are very similar. Red circles indicate pairs producing offspring with female-biased sex ratio, orange diamond labels the pairs with unbiased sex ratio, whereas blue squares indicate pairs producing offspring with male-biased sex ratio.

In order to investigate the potential reason for skewed family sex ratios repeated single pair crossing was carried out. Nineteen breeding pairs were crossed twice on different occasions and their offspring were raised at similar, but not identical conditions (i.e. ambient water temperature ranging between 27–29°C, variable densities and amount feed). Based on the sex ratio of the first set of clutches, the breeding pairs were divided into three groups (Table 1): female-biased group (ten pairs; less than 40% males in their offspring), unbiased group (three pairs; 40–60% males in their offspring) and male-biased group (six pairs; more than 60% males in their offspring). In the female-biased group, the difference in mean male percentages for the first and second batches was 1.3%. The biggest difference produced by a female-biased pair was 15.4% (mating pair 16). Three pairs were assigned to the unbiased group and the mean difference between their 1^st^ and 2^nd^ cross was 6%. In the male-biased group, mating pair 6 showed an unusually big, 25.2% drop in the sex ratio (from 82.9% to 57.7% males) that was 1.6 fold higher than the second highest change and 3.7 fold higher than the mean of the rest. We decided to remove this pair from the comparison and used data for the remaining five pairs only, where the mean difference in the male percentages for the first and second batches was 0.2%. The biggest difference produced by a male-biased pair was 15.1% (mating pair 3). Overall, we observed very similar offspring sex ratios between the first and second crosses from the same breeding pair indicated by the high R^2^ value of 0.8985 (Fig. 2). The fact that sex ratios of different batches of offspring from the same breeding pair were very similar suggests that sex in zebrafish is heritable, whereas wide-ranging sex ratios across the families point towards a complex genetic trait.

**Table 1:** The percentage of males from repeated single pair mating of 19 randomly selected zebrafish pairs.

Mating Pair	Cross (male %)	
	1st	2nd
*Male-biased offspring*		
1	88.1	86.4
2	86.7	79.0
3	75.4	60.3
4	71.0	84.2
5	68.3	78.8
6*	82.9	57.7
*Mean* +	*77.9*	*77.7*
*Unbiased offspring*		
7	57.1	68.6
8	51.2	52.5
9	44.4	49.6
*Mean*	*50.9*	*56.9*
*Female-biased offspring*		
10	37.5	36.7
11	31.8	27.8
12	28.1	19.3
13	22.4	24.7
14	19.4	28.2
15	19.1	15.5
16	18.5	33.9
17	13.1	14.5
18	10.0	15.4
19	0.0	8.0
*Mean*	*25.2*	*26.6*

* Removed from further analysis.

+ Does not include mating pair 6.

### Enhancement and maintenance of sex-biased lines through multiple generations by full-sib selective breeding

This experiment was carried out with the aim to determine if sex-biased ratios in lines can be maintained or increased by selecting for breeding pairs that produced brood with highly skewed sex ratios through several generations.

A total of 5 lines were established and followed through two to four generations. Several lines were split into sub-lines that were later split further depending on the sex ratios resulting from the multifactorial crosses. Altogether, offspring from 26 fourth generation families were grown to maturity and sexed (Fig. S1). In two families, we managed to generate an all-male offspring in the F3 generation (Fig. S1), whereas our efforts to generate all-female offspring were unsuccessful.

**Figure 3:**
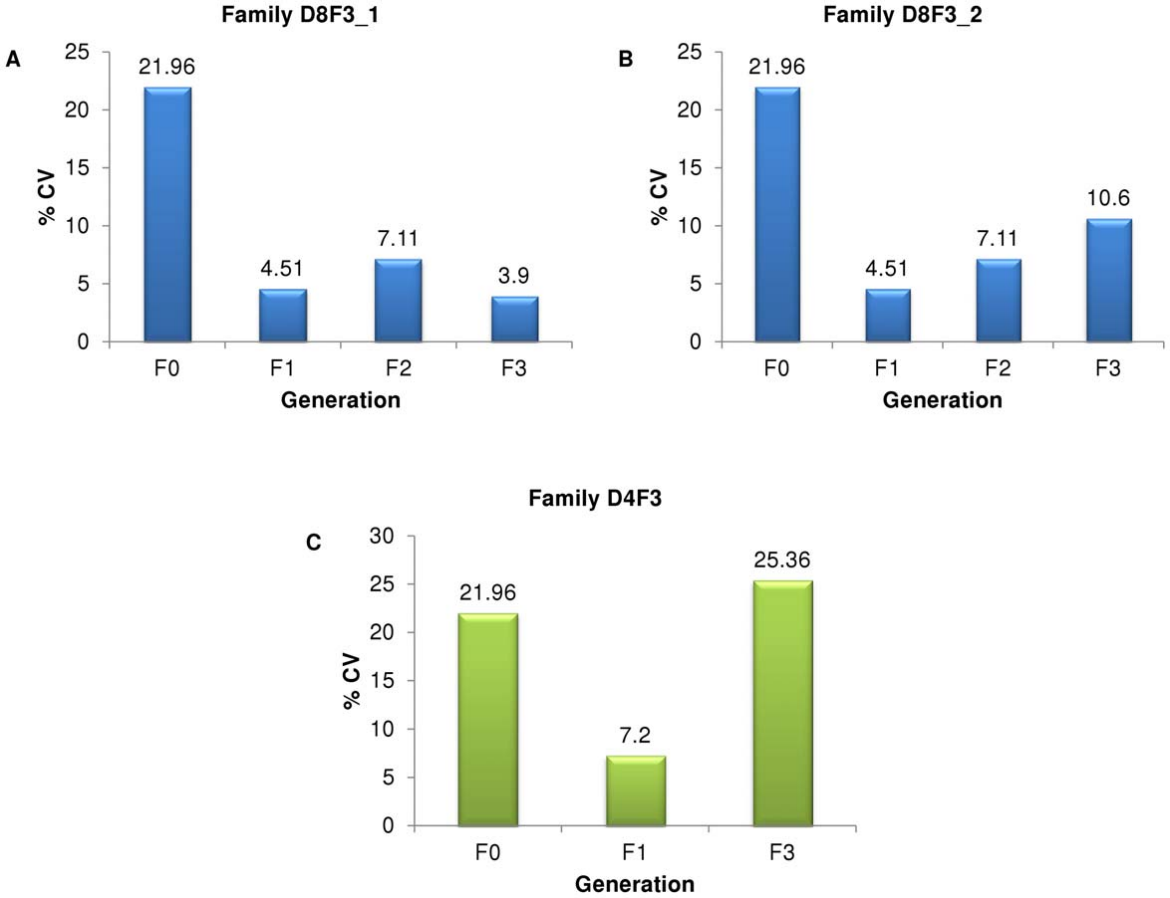
Coefficients of variation for each generation family sex ratios show selection effect on sex ratio. Panels A & B: For both families, CV for the F_0_ generation (unselected) was more than two-folds higher than those for the subsequent generations, which underwent selection. Panel C: In the control experiment, after selecting for pairs that produced high proportion of males at F_0_ generation, CV for F_1_ generation family sex ratio decreased by about three-folds. However, when selection pressure was removed at F_2_ generation by performing a random mass cross, CV for F_3_ generation family sex ratio returned to a level similar to that of unselected F_0_ generation.

Here, we describe two male-biased families that we managed to maintain for two generations through selection from a single line and split in the third generation (Fig. S1). The F_3_ mean sex ratios of the two families were 96.8% (family D8F3_1) and 93.3% (family D8F3_2) males. We then analyzed the family sex ratio variation for each generation by calculating coefficients of variation (CV). It was observed that after selection the family sex ratio CV decreased at least two-folds when compared to the F_0_ generation (Fig. 3A & B). To verify that the decrease was due to selection pressure, we performed a control experiment with the D4F3 family by doing a mass cross for the F_2_ generation without any selection (Fig. 3C). The control family sex ratio CV for the F_0_ generation was 21.96% and after selection the F_1_ generation family sex ratio CV was 7.2%, about three-fold lower. However, after F_2_ mass cross the family sex ratio CV of F_3_ increased to 25.36%. The “bouncing back" of F_3_ family sex ratio CV to a level similar to F_0_ indicates that selection pressure was indeed maintaining the highly skewed family sex ratio. This indicates that zebrafish sex is a genetic trait and the fact that we were able to keep highly skewed sex ratios - and even eliminate one of the two sexes in some cases - suggests the absence of a strong effect by sex chromosomes on sex determination.

### FluoMEP assay identified sex-linked DNA markers from guppy and rosy barb, but not from zebrafish

FluoMEP assay was used to search for DNA markers tightly associated with sex from three different fish species' genomes. The first species was the guppy (*Poecilia reticulata*) which has XX/XY sex chromosomes [56]. Altogether, 144 different primer combinations utilizing the same common motif primer were tested. They yielded three male-specific sex markers (Fig. S3A), that showed 100% agreement with phenotypic sex in eight individuals tested (data not shown). Next, we screened the genome of rosy barb (*Puntius conchonius*) that is also known to have XX/XY sex chromosomes [57], [58], with 386 primer combinations (based on two common primers) and obtained two male-specific sex markers (Fig. S3B). When tested on eight individuals, the sexing efficiency of the two markers was also 100% (data not shown). These results demonstrated that FluoMEP is able to isolate sex-linked DNA markers from fish genomes with substantial differences between the male and female genomes.

In order to search for sex-linked DNA markers in zebrafish, we used a total of 258 FluoMEP primer combinations (based on 29 common primers) to screen pooled male and female zebrafish genomic DNA samples. However, no sex-linked DNA marker was found suggesting that there are no substantial differences between zebrafish male and female genomes.

### No universal sex-linked CNV was detected in four zebrafish families by aCGH

**Figure 4:**
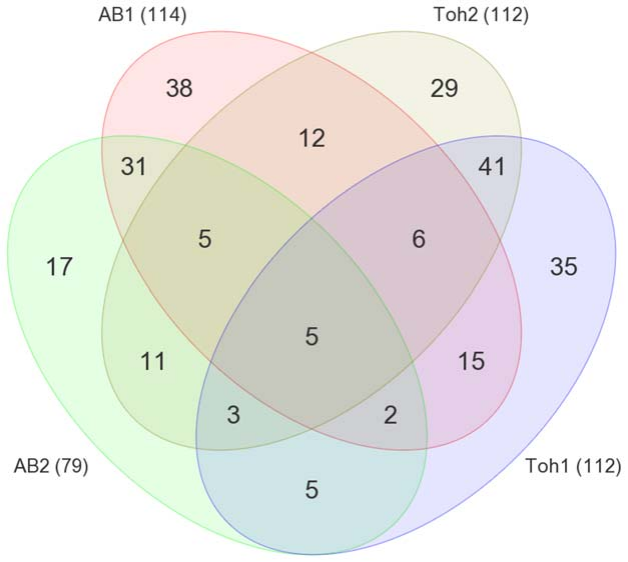
Comparative analysis of CNVRs in four zebrafish families. Out of 255 CNVRs detected, only five were present in all four families tested, however, those common CNVRs have not shown any association with sex. The number of CNVRs detected for each family is indicated in the bracket.

We continued our investigation for sex-linked differences at the genome level by aCGH. We used a custom-designed oligonucleotide microarray containing 120,000 probes covering the assembled zebrafish genome (Zv7). By testing samples from two families each of the AB and Toh strains, a total of 255 CNV regions (CNVRs) were detected (Fig. 4). Among them, 64 CNVRs were present in both strains, 105 were unique to the Toh strain and 86 were present only in the AB strain. Five CNVRs were common to all the four families screened (Fig. 4). As we expected that a sex-determining chromosomal region would be present in all strains, we analyzed the five common CNVRs on individuals, but none of them turned out to be inherited in a sex-linked pattern (Fig. S4).

**Figure 5:**
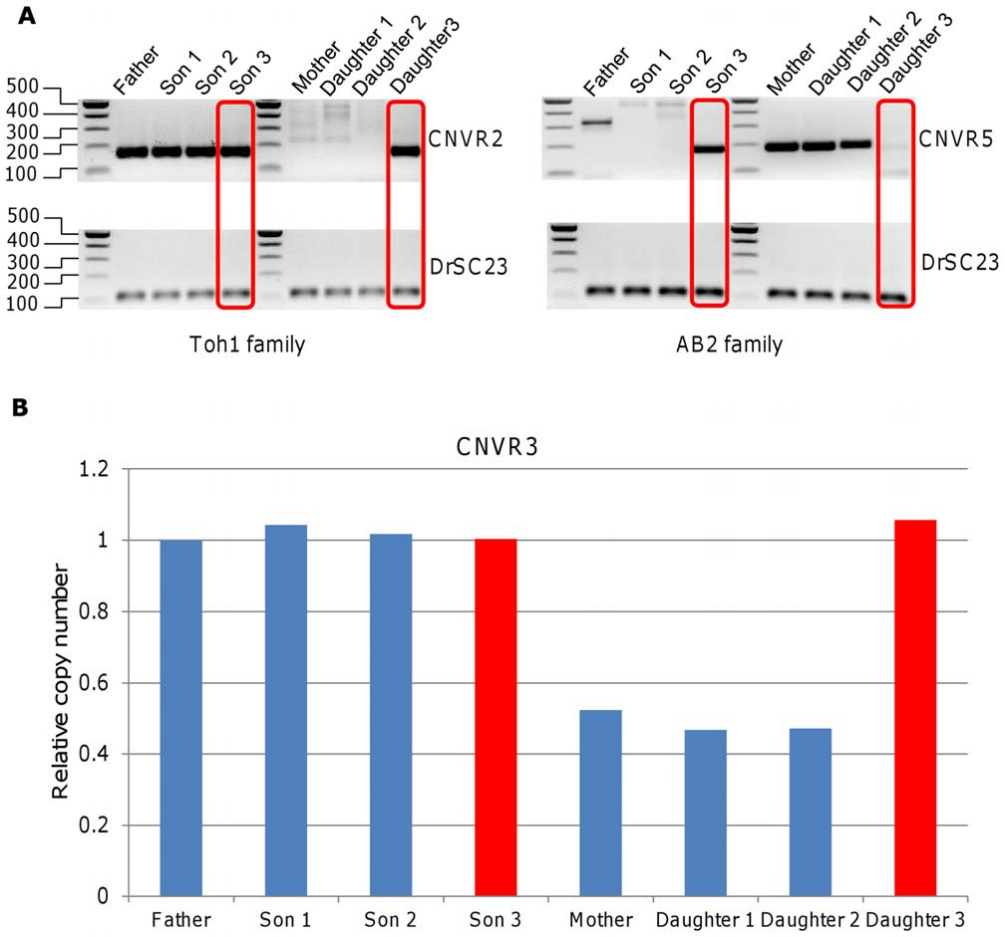
PCR-based validation of aCGH results that showed apparent family specific sex-linked inheritance pattern confirms that none of the three CNVRs analyzed are sex-linked. **A**) The lack of sex-linkage for CNV regions 2 and 5 as confirmed by PCR. Size of the amplified fragments for CNVR2 and CNVR5 are 157 bp and 183 bp, respectively. CNVR2 was present only in males from the Toh1 family (Father and Son 1 and 2), while CNVR5 was only seen in female samples from the AB2 family (Mother and Daughter 1 and 2). As they showed a family-specific, sex-linked pattern, additional offspring (one son and one daughter; red boxes) were used for the validation. Upon further validation, CNVR2 and CNVR5 were found not to be sex-linked. **B**) CNV region 3 could only be validated by real time qPCR. As the three female samples from Toh2 family used for aCGH showed a loss with reference to the father's genome, additional offspring (one son and one daughter; red bar) were used for validation. Further validation also showed that this is not a sex-linked CNVR.

Additional five CNVRs showed apparent family-specific sex linkage (Table 2) and were further analyzed by PCR-based methods with additional two offspring individuals (one male and one female) from the same families. Multiple primers were designed for the first two CNVRs, but failed to yield a PCR product, presumably due to differences between the Zv7 genome assembly used for the probe design and Zv8 used for the analysis of results. Two of the remaining three CNVRs were found not to be sex-linked by PCR (Fig. 5A), while the last one was found not to be sex-specific by real time quantitative PCR (qPCR; Fig. 5B). In fact, none of the additional offspring individuals analyzed did show sex-linked inheritance pattern for any of these three markers. Therefore, we concluded that no family-specific, sex-linked CNVR was identified from the four zebrafish families analyzed.

**Table 2:** CNV regions selected for further validation due to their apparent association to sex based on preliminary aCGH.

	Chromosome	Start	End	Length (Kb)	Family	Copy number
CNVR1	1	56,170,986	57,093,117	922	AB2	Gain
CNVR2	7	664	308,167	308	Toh1	Loss
CNVR3	8	30,107,209	30,331,905	225	Toh2	Loss
CNVR4	8	43,154,384	43,205,453	51	AB2	Gain
CNVR5	8	47,481,469	47,612,299	131	AB2	Gain

### Rearing density has a limited effect on sex ratios in zebrafish

**Figure 6:**
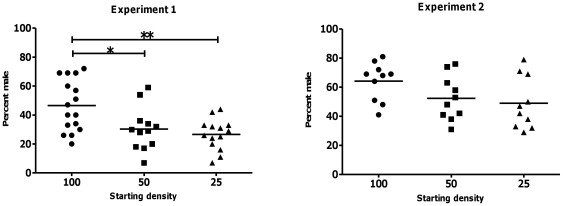
High rearing densities yield higher male to female sex ratios compared to lower ones. Two individual experiments were performed consisting of forty-four populations for experiment 1 and thirty populations for experiment 2. The final percentage of males was assayed for each population and the averages for each population group, denoted by the starting density, were calculated. For each experiment the overall sex ratios varied, but both showed about a twenty percent increase in male percentage in populations with starting densities of 100 fish per 1.5 liters compared to populations with starting densities of 50 or 25 fish per 1.5 liters. Each datapoint represents the percentage of males for a given parental pair, whereas the horizontal line indicates the mean male ratio.

Density has been shown to affect sex ratios in some fish species (see [59] for review). To ask whether rearing density influences sex determination in zebrafish, we raised groups of zebrafish at three different densities from 5 dpf to adulthood and assayed the sex ratios of the resulting adults. The three groups had starting densities of 100, 50, and 25 larvae per 1.5 liters of water, respectively. Two independent experiments were performed that varied slightly in their design (see Materials and Methods) yet resulted in similar outcomes with respect to relative sex ratios across different rearing densities. However, in each experiment, we observed wide-ranging sex ratios at all the tested starting densities (Fig. 6). This profile was similar to that observed in the breeding experiment (see Fig. 1). We found that zebrafish reared at high density had approximately twenty per cent more males on average than those raised at middle or low densities indicating a modest effect of large differences in rearing density on sex determination in zebrafish (Fig. 6).

As the time window in which sex determination occurs is not well defined, we wanted to account for potential changes in population densities due to larval or juvenile death in the above experiments. The number of fish was counted every 10 days beginning on day 10 post fertilization. The highest degree of lethality was typically between 10 and 20 dpf, which corresponded to the period at which both food and water regimes were altered (see Materials and Methods). After 20 dpf, limited loss was observed in most populations and after 30 dpf most individuals survived (Table S1 and Fig. S2). Despite some larval lethality, the average percentage of dead fish in each density group from experiment 2 did not differ significantly indicating that larval death did not contribute to the observed higher percentage of males in the high density group (Table S1 and Fig. S2).

## Discussion

### Molecular and breeding data suggest a genetic sex determination system without a predominant sex chromosome in zebrafish

Although zebrafish has become one of the prime vertebrate models for developmental biology, its sex determination mechanism is still unknown. Therefore, the primary aim of this project was to find out more about the sex determination of this species. The first question we asked was: does zebrafish use a chromosomal sex determination system?

So far, several cytogenetic analyses were performed on zebrafish karyotypes to search for a size-heteromorphic chromosomal pair, which is a hallmark of CSD with highly differentiated sex chromosomes. However, the accurate assignment of chromosomal pairs is hampered by lack of substantial size differences among the zebrafish chromosomes and their poor staining. Ten teams reported the lack of a heteromorphic chromosomal pair in the zebrafish karyotype [60], [61], [62], [63], [64], [65], [66], [67], [68], [69], while only two publications described the presence of such a pair [70], [71]. Researchers have also tried to look for sex chromosomes in the zebrafish genome by searching for sex bivalent synaptonemal complexes [72] and performing comparative genomic hybridization (CGH) between male and female gDNAs [73]. Negative results from both latter studies – together with the vast majority of cytogenetic data - suggest that zebrafish does not possess heteromorphic sex chromosomes.

Our breeding data also indicate the absence of chromosomal sex determination in zebrafish, whereby the inheritance of a particular chromosome would be the predominant determiner of sex. We observed variable family sex ratios from 62 clutches of offspring from different breeding pairs (Fig. 1). Broods from species that have strong chromosomal sex determination system typically exhibit a narrow range of family sex ratios that do not divert substantially from 1∶1 (male to female; e.g. Nile tilapia [4] and rainbow trout [74]). Moreover, we were able to obtain several strongly male-biased zebrafish families by selective crossing of brooders that produced higher proportion of male offspring over a few generations. In the chromosomal sex determination system, the chance for the occurrence of such sex-biased families would be very low, because the ratio of male and female would tend to ‘bounce back’ close to 1∶1 in the next generation. These breeding data also indicate that zebrafish sex determination is unlikely to be based primarily on sex chromosomes.

To further prove the absence of chromosomal sex determination system, we screened the zebrafish genome for sex-linked differences with molecular tools. The first experiments we performed were a series of comparative FluoMEP assays [39]. After screening through 258 FluoMEP primer combinations, no confirmed sex marker was obtained from zebrafish. On the other hand, sex markers for guppy and rosy barb were detected by using the same method. The latter data prove that the FluoMEP assay is suitable for isolating sex-linked markers from genomes known to contain heteromorphic sex chromosomes. The fact that we were unable to obtain sex-linked markers from zebrafish with the same method provides an additional indication that no substantial differences exist between the male and female genomes. Even if there are sex chromosomes in zebrafish, they will be likely showing limited differences at sequence level and therefore undergo recombination with each other along the majority of their length. In this case, the identification of such sex chromosomes through the analysis of pools generated based on phenotypes would be extremely difficult.

Next, we performed array comparative genomic hybridization (aCGH) on four families of zebrafish. Through the analysis of 120 thousand genomic locations, a total of 255 CNVRs were observed and most showed a pattern of Mendelian inheritance. However, no universal sex-linked CNVR was found among the four families of zebrafish tested. The aCGH results suggest that the possibility for highly differentiated, heteromorphic sex chromosomes in the zebrafish genome is quite low. The caveat of our current aCGH approach is that the assembly of zebrafish genome (Zv8) on the basis of which the probes were analyzed managed to assemble only about 89% of the total sequences obtained [75]. Therefore, there is still a possibility that there are sex-linked CNVRs “hiding" in the remaining 11% of the genome. Furthermore, the probes present on the custom-made oligo array have a median spacing of 10 kb intervals and by setting the window of detection to 5 probes per window allows for a resolution of around 50 kb. This means that any genomic difference with less than 50 kb in length will not be picked up by our aCGH approach. However, we argue that the size difference for most active sex chromosomal pairs will likely exceed 50 kb in length, as in case of the medaka, the only known SD region described from teleosts so far [76]. Recently a high resolution zebrafish CNV map was published by analyzing 80 genomes with 1.4 kb probe spacing CNV array [77]. Although it has higher resolution than our CNV array the study did not performed comparative analysis of the male and female genome.

Considering the combined data of the FluoMEP and aCGH approaches, the majority of the (assembled) zebrafish genome was probed for sex-linked sequences in this study. Although we still cannot completely rule out the presence of a sex chromosomal pair, it is unlikely that a single predominant sex-determining region exists. As our breeding data also do not support the presence of a sex chromosome, we provide a strong case against CSD in zebrafish.

Very recently, a genome-wide association study was performed for the identification of sex determining regions with a SNP array containing over 5,300 features [78]. The authors reported two regions on two separate chromosomes (Chr5 and Chr16) accounting for 16% variance of the trait, providing a direct experimental evidence for a polygenic sex determination system in the zebrafish [78]. These data further strengthen the notion that zebrafish sex is not determined by a sex chromosomal pair.

Our data and results from vast majority of the above studies contradict a recent suggestion that zebrafish has a female dominant (ZZ/ZW) sex determination system [79]. Although the results described in that publication seem to support the possibility of a ZZ/ZW sex chromosome system, their data do not conclusively demonstrate that this mode of sex determination is actually in place. Attempts to identify the genetic factor(s) regulating sex or the proposed sex chromosomes were not made in their study.

### Zebrafish sex is determined genetically

Since molecular and breeding studies failed to identify heteromorphic sex chromosomes or their effect, we next sought to find out if genetic factors are involved in zebrafish sex determination. We performed repeated single pair mating in which 19 randomly selected breeding pairs were bred twice. The environmental factors such as ambient temperature, amount of food given and rearing density were not tightly controlled. Even so, broods derived from the same breeding pair did not exhibit major sex ratio differences between repeated crossings of 18 out of 19 breeding pairs tested (Fig. 2). This indicates that the wide-ranging sex ratios normally observed are most likely due to the parental genotypes. In addition, we showed that sex ratio variation decreases substantially under selective pressure, a strong indication that sex is a genetic trait. Another interesting phenomenon we observed was that after three generations of selection we were able to obtain two all-male families while attempts to produce all-female families were unsuccessful. We do not have an explanation for this difference and we propose that further investigations are needed to elucidate the underlying reasons. Nevertheless, our data show that zebrafish uses primarily genetic sex determination system. As we have also demonstrated that CSD is not likely the mode of sex determination in zebrafish, we propose that a PGSD is in place. Based on our data and the recent aforementioned association study [78], we propose that the number of genes contributing to the sex determination process might be far more than just a handful.

### Polygenic sex determination might be more common among vertebrates than expected

The vast majority of our knowledge about vertebrate sex determination was obtained from species using sex chromosomal systems. On the other hand, over 90% of the fish species analyzed through karyotyping does not show the presence of differentiated sex chromosomes (see [5] for review).

Recently, it was proposed that multiple parallel sex determining pathways are likely to operate in species with CSD and this mode of SD could be extended to species with PGSD as well [80]. In such scenario, both systems might have a more similar regulation than expected, differing only in the location of the factors: all would map onto the sex chromosomes in CSD, whereas in PGSD some (or all) of them would be located on the autosomes. Therefore, analysis of zebrafish and other fish species utilizing the PGSD system could be important for basic research and potentially useful for aquaculture projects as well.

### Environmental factors have limited influence on zebrafish sex ratio

Temperature is the most commonly studied environmental cue for sex determination. It is utilized by many reptile species [18], [81], [82], [83] and some fish species [74], [84]. In animals with temperature-based sex determination (TSD), substantial fluctuations in the environmental temperature will likely cause significant changes in the offspring sex ratio [85]. Two papers reported that the temperature at natural habitat of zebrafish ranges from 26 to 38°C [86], [87]. However, it is believed that 26 to 29°C is the temperature range for normal zebrafish development and rearing them within this range did not result in significant sex ratio changes [85]. It was also observed that exposure to increased temperature (35–37°C) either during early development (5–48 hpf) [88] or between 17–27 dpf [89] resulted in male-biased sex ratio. On the other hand, at our laboratories we observed high mortality if zebrafish larvae were grown at 37°C from the beginning. Therefore, temperature is unlikely to be the primary signal for zebrafish sex determination, but might exert secondary effects on its sexual development.

Rearing density is another environmental cue known to influence sex ratio of some fish species such as the American eel [90]. The exact underlying mechanisms of how rearing density directs sexual development are still unknown. We have tested the effect of rearing density on zebrafish sex, and found a substantial increase of males at high density (100 individuals per 1.5 liters of water). In another study, slow growth rate as a result of limited food supply - usually experienced at high rearing density - had been suggested to influence zebrafish sex differentiation leading to higher percentage of males [91]. Nonetheless, we think that rearing density is unlikely to be the primary determinant for zebrafish sex, as we observed wide ranging sex ratios at all three densities tested. A strong determinant should produce broods of very similar sex ratio. In addition, the response to these environmental factors seems to differ between families indicating that influence of rearing density on sex ratio is most likely conferred by the genotype of the fish.

Another environmental factor that is known to have an effect on zebrafish sex ratio is oxygen level [92]. It was found that under hypoxic conditions there was a reduction of estrogen synthesis leading to an increase of androgen to estrogen ratio which favors male development [93]. However, the decreased oxygen level had only limited effect on the sex ratio of zebrafish leading to higher percentage of males (12.5% differences) [92]. This is unlikely the cause of wide ranging sex ratio observed in the zebrafish.

Published data and our results both seem to suggest that non-extreme environmental factors do not have a major effect on zebrafish sex ratio. No drastic change in sex ratio upon environmental effects experienced at the natural surroundings of the species was observed in any of the studies. Furthermore, response to environmental factors varies among the treatment groups. This indicates that the underlying genotype of each individual is directing sexual development in response to environmental stimulus.

### Conclusions

For this study, we performed classical breeding experiments together with large-scale genomic analyses to show that zebrafish sex is determined genetically with no sign of a chromosomal sex determination system. The characteristics of sex ratios observed in zebrafish were as follows: i) wide variation among different families; ii) strong influence from parental genotypes; and iii) the ability to eliminate one of the sexes by selection. All these features point toward a species without a predominant chromosomal sex determination system [11]. Our in-depth investigation by molecular tools (i.e. FluoMEP and aCGH) also failed to identify any difference between the male and female genomes. Several studies, which investigated environmental impacts on zebrafish sex ratio, found them either to result in limited change or show strong effects outside of the physiological range of the species. This cannot account for the wide-ranging sex ratios among families; hence we reckon that zebrafish does not use a primary environmental sex determination system.

Taken together, the above data indicate either the lack of sex chromosomes in zebrafish or the presence of very weak ones that are frequently over-ridden by strong modifier genes. In our opinion, these two situations are principally the same, as there are several genes distributed throughout the genome with major effects on sexual development in both; therefore we propose that zebrafish sex determination should be considered polygenic.

Earlier, others have indicated the possibility of a polygenic sex determination system for zebrafish based on a single set of experiment each (see e.g. [94] & [78]). Our study adds data obtained by four different methods that all point to a polygenic sex determination system, creating a tipping point in this argument.

## Supporting Information

Figure S1 **Multifactorial selective breeding was carried out over a few generations for five lines of zebrafish to select for pairs that produced a sex-biased family.** All multifactorial crosses were set up using full siblings. The selective breeding process involved selecting for pairs that produce highly biased sex ratio (highlighted by different colour box) then offspring from the selected pairs were used to set up multifactorial crosses in the next generation. This was repeated for a few generations. The two all-male families were from the D8 line (D8F3_1 and D8F3_2). The control family D4F3 had a mass cross performed in the F_2_ generation without selection.
(XLSX)

Figure S2 **Plots of the number of fish present in each population over time.** Each line represents one population, housed in a single tank. Data points of fish counts are represented by diamonds. Populations with higher than the overall average percentage of males are colored orange while those populations with a lower than average male percentage are colored blue. These data are from experiment 1 and 2 shown in Table S1.
(PPTX)

Figure S3 **Sex-linked FluoMEP markers obtained by bulk segregant analysis performed on pooled male and female samples of guppy (** ***Poecilia reticulata).*** A) and rosy barb (*Puntius conchonius;* B). Primer combinations are indicated on the top right corner of the peak profiles. Red boxes indicate the sex-linked markers that were confirmed through individual testing. The remaining differences are false positives that have occurred with similar frequency in both species depicted here, as well as in the zebrafish (not shown).
(PPTX)

Figure S4 **Inheritance pattern of the five CNVRs universal to all four families.** None of them showed sex-linked inheritance pattern. Gain in copy number is indicated by blue box while loss in copy number is indicated by red box.
(PPTX)

Table S1 Data tables for rearing density experiment 1 and 2.
(XLSX)

Table S2 Sequences of all primers used.
(XLSX)
