# Supplementary figures and images for: Polygenic Sex Determination System in Zebrafish
Source: PLoS One. 2012 Apr 10;7(4):e34397. doi: 10.1371/journal.pone.0034397 (PMC3323597; doi:10.1371/journal.pone.0034397)

## Slide 1
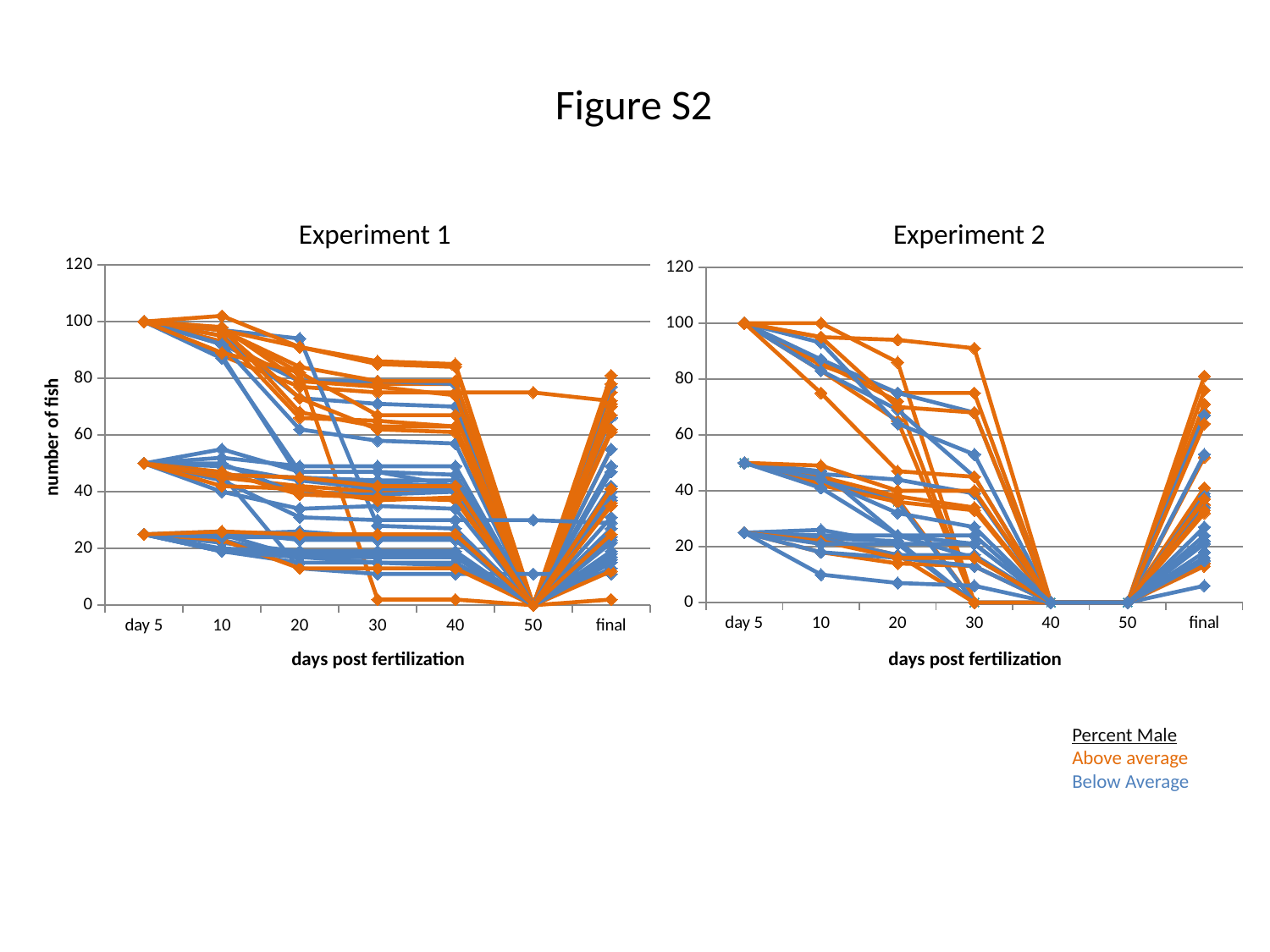

Supplement: Figure S2 — Plots of the number of fish present in each population over time. Each line represents one population, housed in a single tank. Data points of fish counts are represented by diamonds. Populations with higher than the overall average percentage of males are colored orange while those populations with a lower than average male percentage are colored blue. These data are from experiment 1 and 2 shown in Table S1. (PPTX) [file pone.0034397.s002.pptx]

## Slide 1
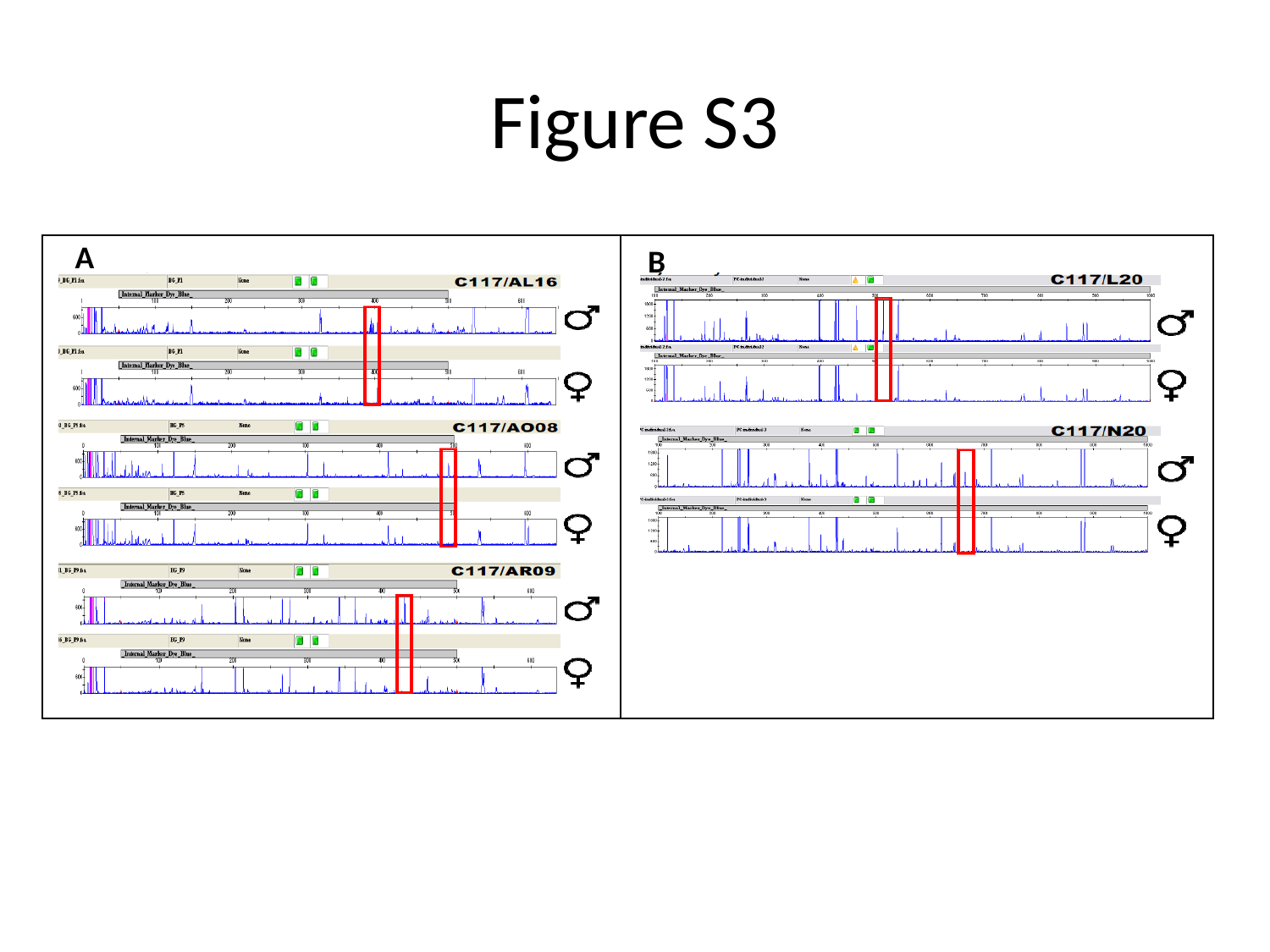

# Figure S3
A
B

Supplement: Figure S3 — Sex-linked FluoMEP markers obtained by bulk segregant analysis performed on pooled male and female samples of guppy ( Poecilia reticulata). A) and rosy barb (Puntius conchonius; B). Primer combinations are indicated on the top right corner of the peak profiles. Red boxes indicate the sex-linked markers that were confirmed through individual testing. The remaining differences are false positives that have occurred with similar frequency in both species depicted here, as well as in the zebrafish (not shown). (PPTX) [file pone.0034397.s003.pptx]
