# Supplementary material for: Polygenic Sex Determination System in Zebrafish
Source: PLoS One. 2012 Apr 10;7(4):e34397. doi: 10.1371/journal.pone.0034397 (PMC3323597; doi:10.1371/journal.pone.0034397)
Supplement: Figure S4 — Inheritance pattern of the five CNVRs universal to all four families. None of them showed sex-linked inheritance pattern. Gain in copy number is indicated by blue box while loss in copy number is indicated by red box. (PPTX) [file pone.0034397.s004.pptx]

## Slide 1
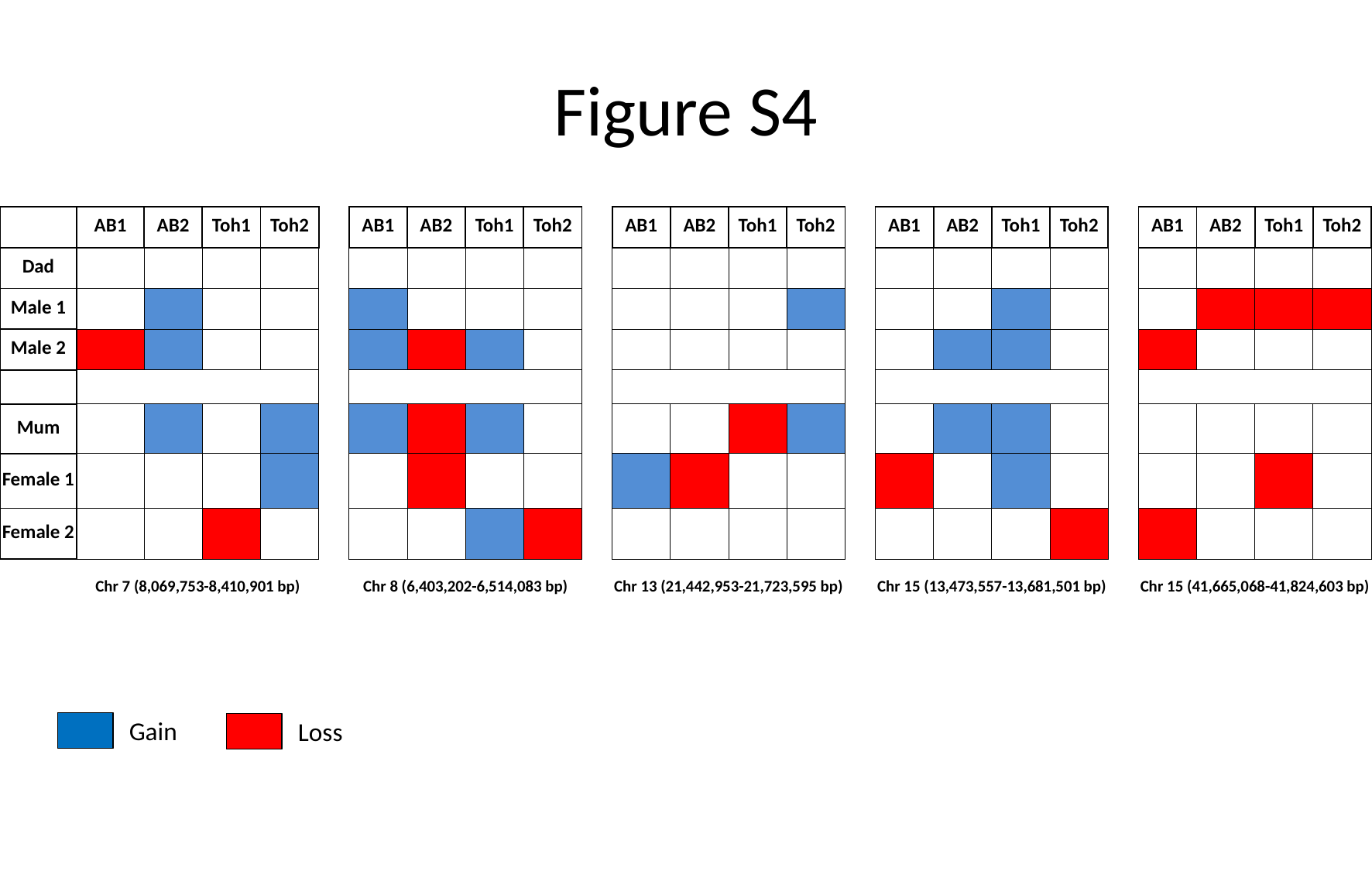

# Figure S4
| | AB1 | AB2 | Toh1 | Toh2 | | AB1 | AB2 | Toh1 | Toh2 | | AB1 | AB2 | Toh1 | Toh2 | | AB1 | AB2 | Toh1 | Toh2 | | AB1 | AB2 | Toh1 | Toh2 |
| --- | --- | --- | --- | --- | --- | --- | --- | --- | --- | --- | --- | --- | --- | --- | --- | --- | --- | --- | --- | --- | --- | --- | --- | --- |
| Dad | | | | | | | | | | | | | | | | | | | | | | | | |
| Male 1 | | | | | | | | | | | | | | | | | | | | | | | | |
| Male 2 | | | | | | | | | | | | | | | | | | | | | | | | |
| | | | | | | | | | | | | | | | | | | | | | | | | |
| Mum | | | | | | | | | | | | | | | | | | | | | | | | |
| Female 1 | | | | | | | | | | | | | | | | | | | | | | | | |
| Female 2 | | | | | | | | | | | | | | | | | | | | | | | | |
| | Chr 7 (8,069,753-8,410,901 bp) | | | | | Chr 8 (6,403,202-6,514,083 bp) | | | | | Chr 13 (21,442,953-21,723,595 bp) | | | | | Chr 15 (13,473,557-13,681,501 bp) | | | | | Chr 15 (41,665,068-41,824,603 bp) | | | |
Gain
Loss
